# Prosthetic overhang is the most effective way to prevent scapular conflict in a reverse total shoulder prosthesis

**DOI:** 10.3109/17453674.2010.538354

**Published:** 2010-11-26

**Authors:** Lieven F de Wilde, Didier Poncet, Bart Middernacht, Anders Ekelund

**Affiliations:** Department of Orthopaedic Surgery and Traumatology, Ghent University Hospital, Ghent, Belgium

## Abstract

**Background and purpose:**

Despite good clinical results with the reverse total shoulder arthroplasty, inferior scapular notching remains a concern. We evaluated 6 different solutions to overcome the problem of scapular notching.

**Methods:**

An average and a “worst case scenario” shape in A-P view in a 2-D computer model of a scapula was created, using data from 200 “normal” scapulae, so that the position of the glenoid and humeral component could be changed as well as design features such as depth of the polyethylene insert, the size of glenosphere, the position of the center of rotation, and downward glenoid inclination. The model calculated the maximum adduction (notch angle) in the scapular plane when the cup of the humeral component was in conflict with the scapula.

**Results:**

A change in humeral neck shaft inclination from 155° to 145° gave a 10° gain in notch angle. A change in cup depth from 8 mm to 5 mm gave a gain of 12°. With no inferior prosthetic overhang, a lateralization of the center of rotation from 0 mm to 5 mm gained 16°. With an inferior overhang of only 1 mm, no effect of lateralizing the center of rotation was noted. Downward glenoid inclination of 0º to 10º gained 10°. A change in glenosphere radius from 18 mm to 21 mm gained 31° due to the inferior overhang created by the increase in glenosphere. A prosthetic overhang to the bone from 0 mm to 5 mm gained 39°.

**Interpretation:**

Of all 6 solutions tested, the prosthetic overhang created the biggest gain in notch angle and this should be considered when designing the reverse arthroplasty and defining optimal surgical technique.

Reverse total shoulder arthroplasty is a well-accepted treatment for the rotator cuff deficient shoulder (Grammont and Baulot 1993, Sirveaux et al. 2004, Werner et al. 2005, Boileau et al. 2006, Simovitch et al. 2007, Wall et al. 2007). However a challenging problem remains: the inferior scapular notching that is located at the inferior pole of the scapular neck (Sirveaux et al. 2004, Werner et al. 2005, Simovitch et al. 2007, Wall et al. 2007, Lévigne et al. 2008). It may worsen clinical outcome, especially if classified as extensive (Sirveaux et al. 2004, Simovitch et al. 2007), and may lead to mechanical failure (Nyffeler et al. 2004, Sirveaux et al. 2004, Vanhove and Beugnies 2004, Boileau et al. 2005, Werner et al. 2005, Simovitch et al. 2007). According to the classifications by Sirveaux et al. (2004) and by Nerot (Valenti et al. 2001), the incidence and extent of notching increases with the length of follow-up and it has been reported to occur in half to nine-tenths of cases with up to 7 years of follow-up (Valenti et al. 2001, Sirveaux et al. 2004, Vanhove and Beugnies 2004, Boileau et al. 2006, Lévigne et al. 2008, Kalouche et al. 2009).

The notch develops as a result of mechanical conflict between the medial border of the humeral implant and the inferior rim of the glenoid (Valenti et al. 2001, Delloye et al. 2002, Boileau et al. 2006), which results in polyethylene wear. This may cause local osteolysis (Valenti et al. 2001, Nyffeler et al. 2004) with progression of the notch (Vanhove and Beugnies 2004). This is supported by the finding of polyethylene wear particles in the pseudomembrane in the osteolytic area (Roberts et al. 2007).

Lévigne et al. (2008) showed that the position of the baseplate influences scapular notching. A superior positioning of the baseplate and a valgus tilting increase the risk of notching. The current surgical practice recommends placing the glenoid component as low (as distal) as possible (Nyffeler et al. 2005, Boileau et al. 2006).

We evaluated the effect on notching of using 6 different positions and designs of the implants.

## Material and methods

We made a 2-D computer model of a scapula. Using the anatomical data from 200 investigated scapulae according to Middernacht et al. (2008), an average shape of an anterior-posterior view of the glenoid cavity, the infraglenoid tubercle, and the lateral border of the scapula was built using an Excel file. The relevant parameters of this model are: the length of the scapula, the angle between the glenoid plane and the scapular pillar, the distance between the center of rotation and the inferior glenoid rim, the distance between the center of rotation and the glenoid plane (lateralization), the diameter of the glenosphere, the downward inclination of the glenosphere, the implant neck shaft angle, and the depth of the conforming PE cup.

A reverse total shoulder prosthesis was inserted in this model so that the position of the glenoid component could be changed, as well as design features such as size of glenosphere and center of rotation. On the humeral side, the depth of the polyethylene insert as well as the neck shaft angle could be varied.

**Figure 1.**
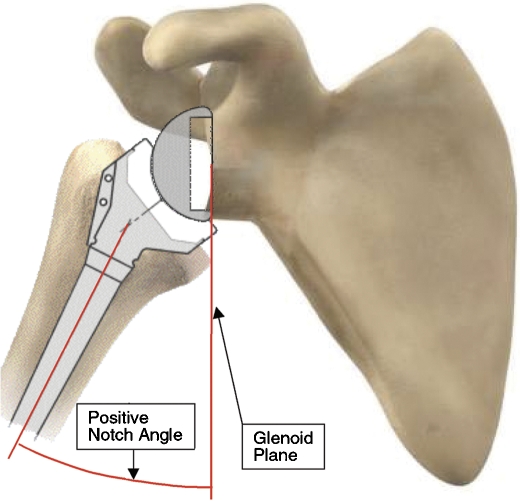
Delta CTA prosthesis build in the average anatomical model based on 200 measured scapulae, with specific interest in the shape of the glenoid cavity, the infraglenoid tubercle, and the lateral border of the scapula. The prosthesis has a flat baseplate situated as distally as possible to ensure full bony coverage of this baseplate with the inferior screw surrounded by minimum 2 mm bone, and a glenosphere 36 mm with a standard polyethylene cup. The notch angand le is the maximum adduction angle before a conflict arises between the PE cup and scapular pillar.

With this model, it was possible to calculate the adduction angle in the scapula plane between the humerus and a vertical line parallel to the glenoid plane, which is the plane formed by the rim of the inferior quadrants of the glenoid (Harman et al. 2005), when a conflict between the polyethylene cup and the scapular pillar occurred. This angle was defined as the notch angle. A positive value means that conflict occurs before the humerus reaches the vertical position. A negative value means that the humerus can be adducted further than the vertical position (Figure 1).

In addition, it was possible to determine where the conflict between the humeral component and the scapula would appear on the scapular pillar and also whether the conflict was with the inner or the outer PE cup diameter.

**Figure 2.**
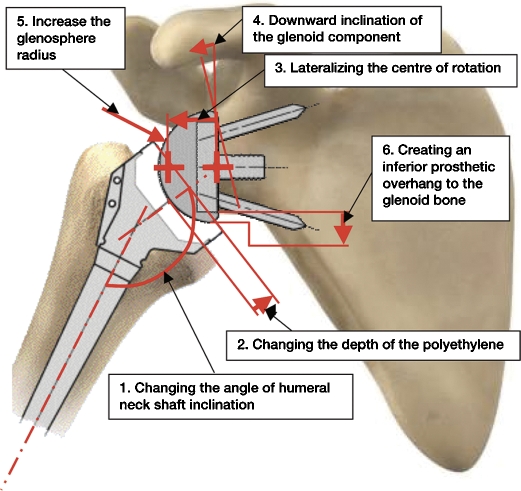
The parameters studied: 1. change in the angle of the humeral neck shaft inclination; 2. change in the depth of the polyethylene cup; 3. lateralization of the center of rotation; 4. downward glenoid inclination; 5. increase in glenosphere radius; 6. creation of an inferior prosthetic overlap with the glenoid bone.

We used the Delta-CTA prosthesis in the computer model, but this design was altered when parameters such as neck-shaft angle, lateralization of the center of rotation, cup depth, and size of glenosphere were evaluated (Figure 2). If a change in one of the tested parameters resulted in an increased ability to adduct the humerus before conflict occurred, it was defined as a gain in notch angle and was expressed in degrees.

## Results

**Figure 3.**
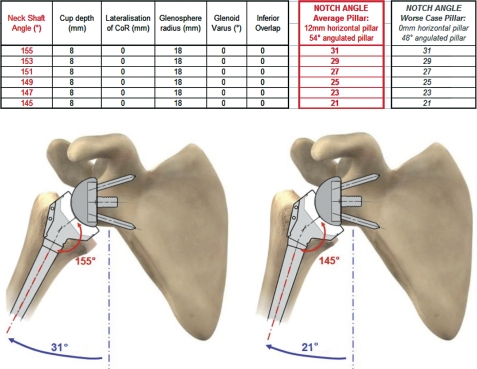
Influence of neck-shaft inclination on the notch angle. (Simulation of maximal adduction in average scapular morphology and in worse-case scapular anatomy: no horizontal pillar. Images of average scapular morphology).

**Figure 4.**
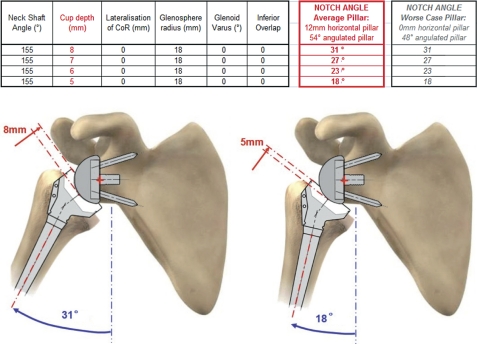
Influence of reduction of cup depth on the notch angle. (Simulation of maximal adduction in average scapular morphology and in worse-case scapular anatomy: no horizontal pillar. Images of average scapular morphology).

A change in humeral neck shaft inclination from 155° to 145° resulted in a gain in notch angle of 10° (31° to 21°) (Figure 3). A change in cup depth (a decrease in prosthetic contact area) from 8 mm to 5 mm resulted in a maximum gain in notch angle of 12° (from 31 to 19°) (Figure 4).

**Figure 5.**
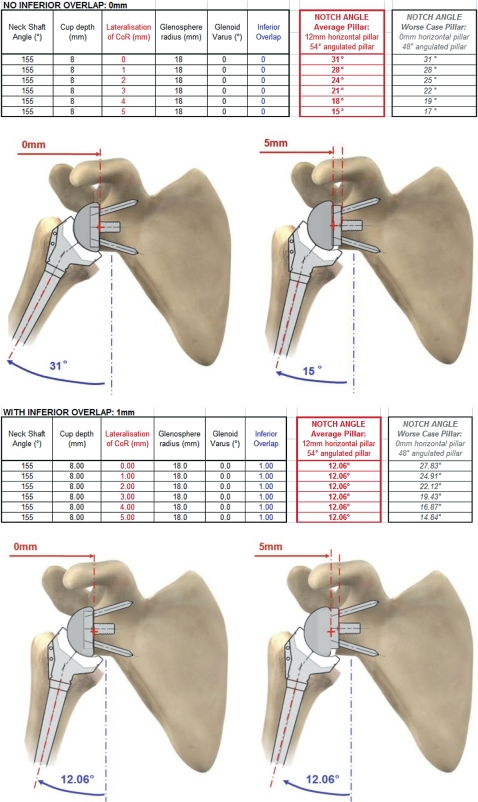
Influence of lateralization on the notch angle. 2 cases with or without inferior overhang. (Simulation of maximal adduction in average scapular morphology and in worse-case scapular anatomy: no horizontal pillar. Images of average scapular morphology).

With no inferior prosthetic overhang, a lateralization of the center of rotation from 0 mm to 5 mm resulted in a maximum gain in notch angle of 16° (from 31° to 15°). More lateralization resulted in increased gain in notch angle. However, with an inferior overhang of only 1 mm there was no effect of lateralizing the center of rotation (12° notch angle for all degrees of lateralization) (Figure 5).

**Figure 6.**
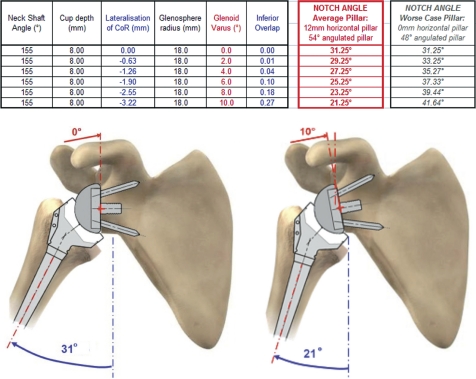
Influence of downward glenoid inclination on the notch angle (called “glenoid varus” in the spreadsheet). (Simulation of maximal adduction in average scapular morphology and in worse-case scapular anatomy: no horizontal pillar. Images of average scapular morphology).

**Figure 7.**
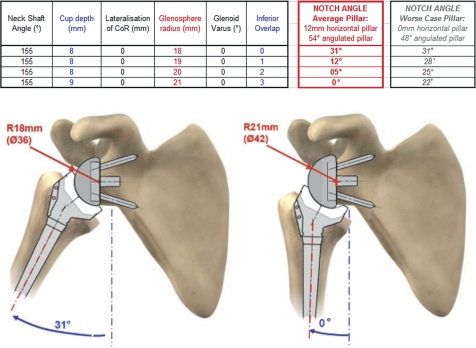
Influence of glenosphere radius on notch angle. (Simulation of maximal adduction in average scapular morphology and in worse-case scapular anatomy: no horizontal pillar. Images of average scapular morphology).

Downward glenoid inclination of 0 to 10° resulted in a gain in notch angle of 10° (from 31° to 21°) with 1 degree of gain for each degree of inclination (Figure 6). A change in glenosphere radius from 18 mm to 21 mm resulted in a gain in notch angle of 31° if the cup depth increased with the glenosphere radius in order to keep the same joint stability (Figure 7). The increase in glenosphere radius had no effect in itself on the notch angle, but it resulted in an increased prosthetic overhang to the bone of up to 3 mm (from a radius of 18 mm to 21 mm).

**Figure 8.**
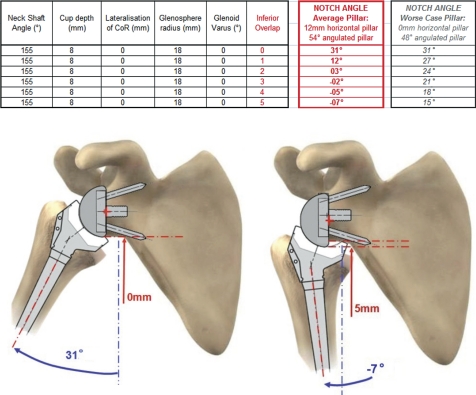
influence of inferior prosthetic overhang on the notch angle. (Simulation of maximal adduction in average scapular morphology and in worse-case scapular anatomy: no horizontal pillar. Images of average scapular morphology).

A prosthetic overhang to the bone from 0 mm to 5 mm resulted in a maximum gain in notch angle of 39° (31° to –8°) (Figure 8).

**Figure 9.**
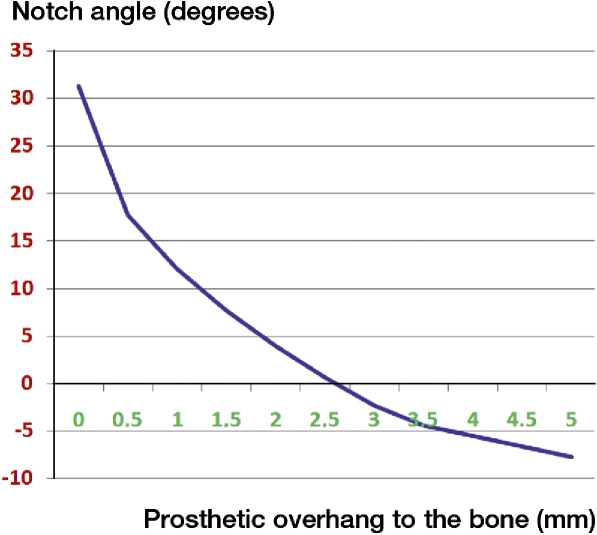
Exponential relationship between the inferior prosthetic overhang and the notch angle.

All relationships were linear—except for the inferior prosthetic overhang, which was exponential. The biggest reduction in the notch angle occurred in the first 2 mm of inferior prosthetic overhang (Figure 9).

## Discussion

The recent literature has shown that prosthetic design parameters of the reverse total shoulder prosthesis are related to the inferior glenoid impingement, which creates the inferior scapular notching phenomenon. In any attempt to minimize notching by changing the prosthetic design, it is important to consider the basic principles introduced by Grammont and Baulot (1993), which result in a very low incidence of loosening of the glenoid component (Gutiérrez et al. 2008a, Roche et al. 2009). Two of the most important principles are the placement of the center of rotation of the shoulder joint, which is the center of the glenosphere, at the glenoid bone-prostheses interface and the implantation on the humeral side of a congruent polyethylene humeral cup, with a non-anatomic inclination of 155° (De Wilde et al. 2005, Boileau et al. 2006).

Changing of the neck-shaft angle by 10º from 155º to 145º resulted in a gain in notch angle of 10º. The decrease in inclination of the humeral prosthetic component from the original 155° as proposed by Grammont and Baulot (1993) decreased the notch angle by as many degrees as the change in inclination. Of course, this reduction in the neck shaft inclination of the humeral prosthetic component reduces the range of movement superiorly (the gain inferiorly = the loss superiorly) (Guttiérrez et al. 2007b, Roche et al. 2009). The actual inclination angle can be influenced surgically by cementing the humeral component in downward or upward inclination by undersizing the humeral component, or by using a short stem or stemless design. However, this also results in reduced stability (Gutiérrez et al. 2008b, Gerber et al. 2009, Roche et al. 2009) and therefore we consider that a change in neck-shaft angle is not recommendable.

A change in cup depth of 3 mm leads to the same concern. A gain in notch angle of 12º was seen, but the price to pay is reduced stability due to less prosthetic contact area (De Wilde et al. 2004, Gutiérrez et al. 2008b, 2009, Gerber et al. 2009, Roche et al. 2009). However, a reduced cup depth allows greater range of motion.

Our findings confirm the work of Nyffeler et al. (2005): a downward glenoid inclination reduces the inferior impingement. The gain in notch angle was the same as the degrees of downward glenoid inclination of the base plate (degrees reamed by the surgeon). However, this reaming removes some of the hard subchondral bone plate of the glenoid, which may weaken the fixation of the glenoid component. Furthermore, on the worst-case scapula (no horizontal pillar), the downward glenoid inclination increased the notch angle. On the other hand, downward glenoid inclination combined with a lateralized center of rotation of the glenosphere decreased this lateralization. Downward glenoid inclination of the glenosphere, by reaming away bone in an inferior tilted way, medializes the center of rotation, which may have a detrimental effect of the forces of the remaining rotator cuff (Boileau et al. 2006). This appears to contrast with the findings of Kontaxis and Johnson (2009) who reported a more important gain in notch angle for downward glenoid tilt (3º). This can be explained by the inferior prosthetic overhang of 2.5 mm that was included in their bony model. Thus, downward glenoid inclination is not recommended to prevent inferior scapular notching.

Lateralization of the center of rotation has been advocated to reduce notching and to improve the adduction of the shoulder (up to 15°) (Gutiérrez et al. 2008 a, b). It can be achieved by changing the design of the glenosphere. However, when the center of rotation is lateralized, the load on the glenoid component increases and this may result in an increased incidence of glenoid component loosening due to a “rocking horse” phenomenon (De Wilde et al. 2004, Hartman et al. 2005, Boileau et al. 2006, Gutiérrez et al. 2007 a, b, Roche et al. 2009). Furthermore, we found that a prosthetic overhang of 1 mm or more completely eliminated the effect of lateralization of the center of rotation on the notch angle. Recently, the BIO-RSA concept was introduced by Boileau (personal communication). To avoid increased load on the glenoid component when the center of rotation is lateralized, they placed a bone graft from the humeral head on the glenoid with the same diameter as the base plate. According to Boileau et al. (2008) and Kalouche et al. (2009), this would reduce notching and keep the center of rotation at the bone-implant interface (Boileau et al. 2005). However, we found that the effect of such a bone graft is mainly due to the fact that the bone graft has a diameter smaller than the glenosphere, thus creating a prosthetic overhang.

Lateralization alone is therefore not important in reducing inferior impingement and the risk of notching. The effect on notch angle is completely eliminated if the design of the prosthetic system allows the surgeon to create an inferior prosthetic overhang (Chou et al. 2009). However, lateralization may have other effects such as influencing (increasing/decreasing) the tension of the remaining rotator cuff (Boileau 2008) and creating a more favorable cosmetic contour of the shoulder (Kalouche et al. 2009). Furthermore, when the scapula has no neck the BIO-RSA converts such a scapula to a more “favorable” shape.

An inferior prosthetic overhang of 5 mm resulted in a gain in notch angle of 39º. A prosthetic overhang therefore seems to be an important factor to consider when performing a reverse shoulder arthroplasty. A low placement of the base plate is advocated, since it increases the ability of the surgeon to achieve an inferior overhang. Prosthetic overhang may also be facilitated by design factors such as size of glenosphere, polyaxial screws, and eccentric glenospheres. However, too much overhang may “overtension” the deltoid muscle, stretch the brachial plexus, and increase the risk of fatigue fracture of the acromion or spine of scapula (Debeer and Robyns 2005). The biggest gain in notching is seen with the first 2 mm of overhang (27°), so there is no need to try to achieve the maximum amount of overhang during surgery.

A limitation of our study is that we used a 2-D model of an average scapula without taking into account the muscles or soft tissues. Different shapes of the inferior part of the scapula influence the notch angle and the ability of different designs to create an inferior overhang. Even with the “worst-shaped” scapulae, scapulae with no neck, the notch angle was most effectively reduced by creating an inferior overhang. The smaller the horizontal pillar, the smaller the difference. If no horizontal pillar exists, 2 mm of lateralization decreases the notch angle by 6° whereas 2 mm of inferior overlap results in 8º. In a more realistic case, in a scapula with a small horizontal pillar of 5 mm (average = 12 mm), the notch angle will decrease by 6° for 2 mm of lateralization and by 20° for 2 mm of inferior overlap.

**Figure 10.**
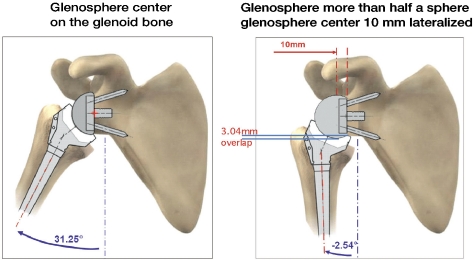
Explanation of the dilemma of Gutiérrez: a prosthetic overhang can also be created by the prosthesis if the center of rotation is lateralized and if the glenosphere is more than half a sphere.

Comparing the different solutions, one must realize that our computer model can only generate theoretical absolute values, which clearly demonstrates that inferior prosthetic overhang is most effective in preventing the conflict. No statistical test can be applied to prove this superiority, because the absolute values are the proof. In this respect, our study shows that the most important parameter to prevent notching is to create a prosthetic overlap in all types of reverse total shoulder prosthesis, by design and surgical positioning. This may be contradictory to the findings of Gutiérrez et al. (2008 a), but it can be explained by the fact that their study was done with a glenosphere of more than half a sphere (10 mm lateralization for a glenosphere of 36 mm). This design mimics a prosthetic overhang of more than 3.04 mm within the prosthesis, thereby improving the adduction (increase in notch angle) by 33° (Figure 10). This phenomenon could explain our findings showing clearly that the first mm of “overhang” are the most important in preventing the inferior scapular conflict.

One must realize that the conclusions of our study concerning the prosthetic overhang in the scapular plane of the body may also be true for the transverse plane. Similarly to the scapular plane of the body where the overhang creates a gain in adduction, a gain in internal and external rotation can be expected in the transverse plane of the body (Gutiérrez et al. 2008 a, Karelse et al. 2008).
